# Ribociclib in Kombination mit endokriner Therapie verbessert Überleben von prä-/perimenopausalen Mammakarzinom-Patientinnen

**DOI:** 10.1007/s00066-019-01574-y

**Published:** 2020-01-16

**Authors:** David Krug, Alexander Fabian, Jürgen Dunst

**Affiliations:** Klinik für Strahlentherapie, Universitätsklinikum Schleswig-Holstein, Arnold-Heller-Str. 3, 24105 Kiel, Deutschland

## Hintergrund

Die zugelassenen Inhibitoren der Cyclin-abhängigen Kinasen 4/6 (CDK4/6i) Palbociclib, Ribociclib und Abemaciclib verbessern das progressionsfreie Überleben von Patientinnen mit Hormonrezeptor-positivem (HR-pos.), HER2-negativem (HER2-neg.) Mammakarzinom in Kombination mit einer endokrinen Therapie deutlich. Die vorgestellte Arbeit präsentiert Daten zum Gesamtüberleben („overall survival“, OS) aus der MONALEESA-7-Studie.

## Patienten und Methodik

Insgesamt wurden 672 prä- bzw. perimenopausale Patientinnen analysiert, die randomisiert entweder mit einer endokrinen Therapie (ET; Goserelin + Letrozol, Anastrozol oder Tamoxifen) + Placebo oder mit einer ET + Ribociclib (600 mg p.o. für 21 Tage, dann 7 Tage Pause) behandelt wurden. Alle Patientinnen waren aufgrund eines lokoregionär rezidivierten oder metastasierten Mammakarzinoms schon vorher palliativ behandelt worden. Die Patientinnen durften maximal ein palliatives Chemotherapie-Regime vor Einschluss erhalten haben, eine vorangegangene palliative endokrine Therapie war nicht erlaubt. Die Randomisierung wurde nach dem Vorhandensein einer viszeralen Metastasierung, vorangegangener Chemotherapie und der Wahl der endokrinen Therapie (Tamoxifen vs. Aromataseinhibitor [AI]) stratifiziert. Der primäre Endpunkt der Studie war das progressionsfreie Überleben („progression-free survival“, PFS); diese Daten wurden bereits 2018 publiziert. Das mediane Follow-up lag bei 35 Monaten.

## Ergebnisse

Das mediane OS war im Experimentalarm noch nicht erreicht und lag im Standardarm bei 40,2 Monaten, dieser Unterschied war statistisch signifikant (Hazard-Ratio [HR] 0,71; *p* = 0,009073). Die geschätzten 4‑Jahres-Überlebensraten betrugen 70,2 % vs. 46 %. Ein signifikant verbessertes OS wurde nur für Patientinnen gezeigt, die einen AI erhalten hatten, wobei die Subgruppe der mit Tamoxifen behandelten Patientinnen nur ein Viertel des Gesamtkollektivs ausmachte. Die einzige Gruppe, die ein Ergebnis zu Ungunsten des Experimentalarms zeigte, waren Patientinnen mit einem progressionsfreien Intervall von mehr als 12 Monaten nach Ende der vorangegangenen (adjuvanten) endokrinen Therapie. Diese Subgruppe war mit 60 Patientinnen allerdings ebenfalls klein und der Unterschied nicht statistisch signifikant. Der Anteil der Patientinnen, die 42 Monate nach Einschluss noch keine Chemotherapie erhalten hatten, war im Experimentarm mit 65,8 % signifikant höher als im Placeboarm (49 %; HR 0,6). Die häufigsten relevanten Grad-III/IV-Nebenwirkungen waren das Auftreten einer Neutropenie (63,5 % vs. 4,5 %), hepatobiliäreToxizitäten (11 % vs. 6,8 %) und eine Verlängerung des QT-Intervalls (1,8 % vs. 1,2 %)

## Schlussfolgerung

Die Hinzunahme von Ribociclib zur ET erzeugt einen substanziellen klinischen Vorteil.

## Kommentar

In der metastasierten Situation besteht die Erstlinientherapie bei palliativem Behandlungsziel und HR-pos. und HER2-neg. Erkrankung üblicherweise aus einer endokrinen Therapie. Eine klare Indikation zur Chemotherapie besteht laut aktuellen Leitlinien nur bei einer drohenden viszeralen Krise, z. B. aufgrund einer Metastasenleber [1]. Bei einer Vielzahl von Patientinnen mit luminalen Tumoren liegt eine primäre oder sekundäre endokrine Resistenz vor. Vor Einführung der CDKi war bereits die Kombination aus Exemestan und Everolimus als endokrin-basierte Kombinationstherapie in der Zweitlinie verfügbar.

Die CDKi mit den Vertretern Palbociclib, Ribociclib und Abemaciclib erfreuen sich seit der Markteinführung in den vergangenen Jahren, basierend auf einer substanziellen PFS-Verbesserung gegenüber der alleinigen ET in der palliativen Erst- und Zweitlinientherapie, enormer Beliebtheit. Prä- und perimenopausale Frauen waren in den bisherigen Studien unterrepräsentiert gewesen. Die hier vorgestellten Ergebnisse der MONALEESA-7-Studie belegen eindrucksvoll die Effektivität der endokrin-basierten Kombinationstherapie. In der PALOMA-3-Studie konnte mit ET + Palbociclib bei den prä-/perimenopausalen Patientinnen keine OS-Verbesserung nachgewiesen werden [2], wobei der Anteil der bereits mit Chemotherapie vorbehandelten Patientinnen hier erheblich höher war als in der MONALEESA-7-Studie (34 % vs. 14 %). Die Toxizitäten der CDKi, insbesondere die Neutropenie, sind mittlerweile bekannt. Relevant ist dies v. a. wegen der Notwendigkeit regelmäßiger Blutbildkontrollen unter der laufenden Therapie und der damit verbundenen Arztbesuche.

Ein Update der S3-Leitlinie zu den CDKi ist aktuell in Bearbeitung. Bereits seit längerer Zeit ist ein Streit über die Bewertung des Zusatznutzens der CDKi durch das IQWiG und den Gemeinsamen Bundesausschuss entbrannt. Trotz einer hoch signifikanten Verbesserung des progressionsfreien Überlebens und teils auch des Gesamtüberlebens durch Hinzunahme der CDKi zur endokrinen Therapie wurde in der überwiegenden Zahl der analysierten Subgruppen kein Zusatznutzen bescheinigt. Begründet wurde dies u. a. mit den erhöhten Nebenwirkungsraten, dem nicht konsistent gezeigten OS-Vorteil sowie mit Strittigkeiten bzgl. der zweckmäßigen Vergleichstherapie.

Die Ergebnisse im Kontrollarm belegen, dass ein mehrjähriges Überleben für Patientinnen mit metastasiertem HR-pos. und HER2-neg. Mammakarzinom mittlerweile vielfach erreichbar ist. Es sind aktuell zahlreiche weitere systemische Therapieoptionen beim metastasierten, HR-pos./HER-2-neg. Mammakarzinom in Entwicklung. Durch die amerikanische Arzneimittelbehörde FDA wurde der PI3K-Inhibitor Alpelisib für Patientinnen mit PI3K-mutiertem Mammakarzinom aufgrund der PFS-Verbesserung in der SOLAR-1-Studie [3] bereits zugelassen. Auch der PARP-Inhibitor Olaparib ist durch die FDA für Mammakarzinom-Patientinnen mit BRCA-Mutation, unabhängig vom HR-Status, zugelassen worden [4]. Eine neue Kombinationstherapie aus zwei zugelassenen Substanzen wurde kürzlich durch Mehta et al. publiziert [5]. Im Rahmen einer Phase-III-Studie wurde durch die Kombination von Fulvestrant + Anastrozol ein signifikanter OS-Vorteil von 7,9 Monaten gegenüber der Monotherapie mit Anastrozol erzielt, trotz einer Crossover-Rate von 45 %. Im Kombinationsarm traten bei 15 % der Patientinnen Grad-III-Nebenwirkungen auf. Zum Vergleich: In der hier vorgestellten Studie waren es im Ribociclib-Arm allein >60 % Grad-III/IV-Neutropenien.

Aus strahlentherapeutischer Sicht bleibt zu erwähnen, dass es bislang nur wenige Erfahrungswerte zur Kombination von CDKi und palliativer Bestrahlung gibt. Die vorhandenen Fallserien ergaben, abgesehen von der bekannten Hämatotoxizität, keinen Anhalt für eine erhöhte Toxizität [6–9] mit Ausnahme eines Falles einer schweren Enterokolitis nach palliativer ossärer Radiotherapie in Kombination mit Palbociclib [10].

## Fazit

Die CDKi haben das therapeutische Spektrum beim HR-pos./HER2-neg. Mammakarzinom deutlich erweitert. Die präsentierten Daten zeigen eine OS-Verbesserung bei prä-/perimenopausalen Patientinnen durch Hinzunahme von Ribociclib zur endokrinen Therapie. Trotzdem sind die Substanzen, u. a. aufgrund ihrer Hämatotoxizität, nicht unumstritten. Ihr endgültiger Stellenwert wird sich in den kommenden Jahren im Wettbewerb mit den weiteren bereits zugelassenen bzw. in Zulassung befindlichen Substanzen zeigen.

*David Krug, Alexander Fabian und Jürgen Dunst, Kiel*
