# Correction: Ribociclib in Kombination mit endokriner Therapie verbessert Überleben von prä-/perimenopausalen Mammakarzinom-Patientinnen

**DOI:** 10.1007/s00066-021-01779-0

**Published:** 2021-04-14

**Authors:** David Krug, Alexander Fabian, Jürgen Dunst

**Affiliations:** Klinik für Strahlentherapie, Universitätsklinikum Schleswig-Holstein, Arnold-Heller-Str. 3, 24105 Kiel, Deutschland

**Correction:**

**Strahlenther Onkol 2020 196:286–288**

10.1007/s00066-019-01574-y

Der Artikel „Ribociclib in Kombination mit endokriner Therapie verbessert Überleben von prä-/perimenopausalen Mammakarzinom-Patientinnen“ von David Krug, Alexander Fabian und Jürgen Dunst wurde ursprünglich Online First ohne „Open Access“ auf der Internetplattform des Verlags publiziert. Nach der Veröffentlichung in Band 196 Heft 3 pp. 286–288 hatten sich die Autoren für eine „Open Access“-Veröffentlichung entschieden. Das Urheberrecht des Artikels wurde deshalb in © Der/die Autor(en) 2020 geändert.

Dieser Artikel ist jetzt unter der Creative Commons Namensnennung 4.0 International Lizenz veröffentlicht, welche die Nutzung, Vervielfältigung, Bearbeitung, Verbreitung und Wiedergabe in jeglichem Medium und Format erlaubt, sofern Sie den/die ursprünglichen Autor(en) und die Quelle ordnungsgemäß nennen, einen Link zur Creative Commons Lizenz beifügen und angeben, ob Änderungen vorgenommen wurden. Die in diesem Artikel enthaltenen Bilder und sonstiges Drittmaterial unterliegen ebenfalls der genannten Creative Commons Lizenz, sofern sich aus der Abbildungslegende nichts anderes ergibt. Sofern das betreffende Material nicht unter der genannten Creative Commons Lizenz steht und die betreffende Handlung nicht nach gesetzlichen Vorschriften erlaubt ist, ist für die oben aufgeführten Weiterverwendungen des Materials die Einwilligung des jeweiligen Rechteinhabers einzuholen. Weitere Details zur Lizenz entnehmen Sie bitte der Lizenzinformation auf http://creativecommons.org/licenses/by/4.0/deed.de.

